# Expression of FAP-1 by human colon adenocarcinoma: implication for resistance against Fas-mediated apoptosis in cancer

**DOI:** 10.1038/sj.bjc.6602136

**Published:** 2004-10-19

**Authors:** H Yao, E Song, J Chen, P Hamar

**Affiliations:** 1 Department of Oncology, Sun-Yat-Sen Memorial Hospital, Guangzhou, People's Republic of China; 2 Department of Surgery, Sun-Yat-Sen Memorial Hospital, Guangzhou, People's Republic of China; 3 Institute of Pathophysiology, Department of Medicine, Semmelweis University, Nagyvárad tér 4, Budapest H-1089, Hungary

**Keywords:** Fas-associated phosphatase-1, Fas receptor, colon neoplasms, apoptosis, immune escape

## Abstract

Although colon carcinoma cells express Fas receptors, they are resistant to Fas-mediated apoptosis. Defects within the intracellular Fas signal transduction may be responsible. We investigated whether the Fas-associated phosphatase-1 (FAP-1), an inhibitor of Fas signal transduction, contributed to this resistance in colon carcinomas. *In vivo*, apoptosis of cancer cells was detected *in situ* using terminal deoxynucleotidyltransferase-mediated dUTP nick-end labelling (TUNEL). FAP-1, FasR, and Fas ligand (FasL) were detected using immunohistochemistry. *In vitro*, colon carcinoma cells were primarily cultured, and their sensitivity to Fas-mediated apoptosis was evaluated by treatment with agonistic anti-FasR CH11 IgM monoclonal antibody in the presence or absence of synthetic Ac-SLV (serine-leucine-valine) tripeptide. Fas-associated phosphatase-1 expression was detected in 20 out of 28 colon adenocarcinomas. *In vivo*, a positive correlation between the percentage of apoptotic tumour cells and the number of FasL-positive tumour infiltrating lymphocytes was observed in FAP-1 negative cancers, but not in FAP-1-positive ones. Primarily cultured colon cancer cells, which were refractory to CH-11-induced apoptosis, had higher expression of FAP-1 on protein and mRNA levels than the sensitive group. Resistance to Fas-mediated apoptosis in tumour cells could be abolished by Ac-SLV tripetides. Fas-associated phosphatase-1 expression protects colon cancer cells from Fas-mediated apoptosis, and blockade of FAP-1 and FasR interaction sensitises tumour cells to Fas-dependent apoptosis.

Despite the presence of tumour-specific cytotoxic T lymphocytes (CTLs) and natural killer (NK) cells, the immune system fails to contain colon cancers. These malignancies are able to escape immune clearance, and are resistant to cytotoxic activities of host immunonocytes (Halapi, 1998; Beatty and Paterson, 2000; Song *et al*, 2001a). However, the exact mechanisms by which colon cancer cells escape CTL or NK cell-mediated destruction remain poorly understood.

The Fas–Fas ligand (FasL) interacting system has been recognised as a major pathway for CTL- and NK cell-mediated apoptotic cell death (Kägi *et al*, 1994; Lowin *et al*, 1994; Arase *et al*, 1995). When CTLs or NK cells recognise target cells, they become activated and express FasL, which binds to Fas receptors on the surface of the target cells and induce target cell apoptosis (Leithauser *et al*, 1993; Suda *et al*, 1993; Suda and Nagata, 1994; Nagata, 1997). Upon contact with crosslinking anti-Fas antibodies or FasL, the Fas-bearing cells rapidly undergo apoptosis. Although colon cancer cells abundantly express Fas receptors at mRNA and protein level, they are insensitive to FasR-mediated apoptosis (Leithauser *et al*, 1993; O'Connell *et al*, 1998; Grosch *et al*, 2001; Vermijlen *et al*, 2001).

The resistance of most colon cancer cells to FasL-induced apoptosis may explain their capability to escape immune cytolysis (Tillman *et al*, 1998; von Reyher *et al*, 1998). However, the molecular mechanisms of this process are still unknown. Some authors suggest that the FasR resistance of colon cancer cells is based on defects of intracellular Fas signal transduction (O'Connell *et al*, 1998; Vermijlen *et al*, 2001).

Fas-associated phosphatase-1 (FAP-1) is a tyrosine phosphatase, which intracellularly inhibits FasR-mediated apoptosis. By interacting with the cytoplasmic death domain of Fas receptors, FAP-1 acts as a negative switch in the Fas pathway. Introduction of FAP-1 into Fas-sensitive cells blocks FasL-induced apoptosis (Ungefroren *et al*, 2001). Furthermore, FAP-1 overexpression correlates with the resistance of some human malignant cells to Fas-mediated apoptosis, such as pancreatic cancer cells, hepatoblastoma cells, leukaemia T cells and AIDS-associated Kaposi's sarcoma cells (Lee *et al*, 1999a).

In a multistep oral carcinogenesis model, the expression of FAP-1 increased in parallel to the progression towards malignancy (Itakura *et al*, 2000). On the other hand, spontaneous downregulation of FAP-1 expression contributes to the excessive apoptosis of haematopoietic cells in myelodysplastic syndromes (Bernhard *et al*, 2001). Furthermore, interruption of the conjugation between FasR and FAP-1 by a synthetic Serine-Leucine-Valine (Ac-SLV) tripeptide abolished the resistance to FasR-mediated apoptosis in human thymocytes (Song *et al*, 2001b). Thus, it is clear that FAP-1 plays an essential regulatory role in Fas-mediated apoptosis. However, although colon cancers have been found to express FAP-1, its contribution to Fas resistance has not been determined.

Therefore, the present study examined the expression of FAP-1 in colon cancer cells, and investigated whether FAP-1 contributed to the resistance of colon cancer against FasR-mediated apoptosis.

## MATERIALS AND METHODS

### Patients and tissue samples

Tissue samples of human colon adenocarcinomas from 28 patients were collected, with informed consent, after surgical resections performed at Sun-Yat-Sen University of Medical Science, Guangzhou, following a protocol approved by the University Teaching Hospitals ethics committee. None of the patients had received chemo-, radio-, or immuno-therapy before resection. Tumours were well (*n*=9), moderately (*n*=11), and poorly (*n*=8) differentiated including three cases of mucinous adenocarcinomas. Pathological staging followed the rules of the Dukes system, with seven cases of Dukes A (T_1_N_0_M_0_: mucosa only), 12 cases of Dukes B (T_2_N_0_M_0_: into but not through the muscularis propria) and nine cases of Dukes C (T_*n*_N_1_M_0_: locally positive nodes). None of the cases was found to have metastases to distal organs. Tissue samples were used in part for primary cell cultures. The rest was snap frozen in liquid nitrogen and stored at −80°C for immunohistochemical staining.

### Immunohistochemistry

A polyclonal antibody for Fas (C20; dilution 1/50), a polyclonal antibody for FasL (N20; dilution 1/50), and a polyclonal antibody for FAP-1 (C20; dilution 1/50) were obtained from Santa Cruz Biotechnology (Santa Cruz, CA, USA). A monoclonal antibody for leucocyte common antigen CD45 (clone 2B11+PD7/26; dilution 1/70) for the identification of tumour infiltrating lymphocytes (TILs) and a labelled streptavidin–biotin (LSAB) immunohistochemical detection kit were obtained from Dako Corp. (Carpinteria, CA).

Acetone-fixed consecutive cryostat tissue sections (4 *μ*m) of colon cancers were stained immunohistochemically with the above antibodies according to the standard LSAB staining techniques. Briefly, endogenous peroxidase activity was quenched by incubation with 3.0% hydrogen peroxide in methanol for 5 min. Sections were washed in phosphate-buffered saline (PBS) and blocked for 1 h in a washing buffer containing 5% normal goat serum (Sigma Chemical Co, St Louis, MO, USA). The primary antibody was added for incubation overnight at 4°C. After washing in PBS, the sites of primary antibody binding were localised by sequential incubation with biotinylated goat anti-rabbit antibody and then streptavidin conjugated with horseradish peroxidase. After further washing in PBS, diaminobenzidine (DAB; Dako Corp., Carpinteria, CA, USA) was used as a chromogen and sections were lightly counterstained with haematoxylin. As negative controls for Fas, FasL, and FAP-1, the immunising peptide to which the antibodies were raised (Dako Corp. Carpinteria, CA, USA) were added at 1 *μ*g ml^−1^ to the primary antibody as a direct, internal competitive control. As a negative control for CD45, we used nonimmune mouse serum instead of the CD45 monoclonal antibody.

### Assessment of FasL-positive TILs

Tumour infiltrating lymphocytes were identified by CD45 staining in consecutive sections, and those that were adjacent to tumour nests were counted for positive immunohistochemical staining of FasL under high-power microscopy (× 400, more than 30 fields counted per section). The view fields for cell counting were chosen according to a systematic random sampling method. FasL-positive TILs were expressed as cells per field of view (c/FV).

### Detection of *in situ* cell death by terminal deoxynucleotidyltransferase-mediated dUTP nick-end labelling (TUNEL)

Cell death was detected *in situ* in resected tissues by enzymatic labelling of DNA strand breaks with a TUNEL assay (Boehringer Mannheim GmbH, Mannheim, Germany) according to the manufacturer's instructions. Cryostat sections were treated with proteinase K (20 *μ*g ml^−1^ in 10 mM Tris/HCl, pH 7.6) for 30 min. After washing, peroxidase block was similar to that described above. The samples were permeabilised (0.1% Triton X-100 in 0.1% sodium citrate 15 min). For the labelling reaction, a TdT buffer solution (composition: potassium cacodylate 100 mmol l^−1^; cobalt chloride 2 mmol l^−1^; dithiothreitol 0.2 mmol l^−1^; pH 7.2) containing 0.3 U *μ*l^−1^ biotinylated dUTP was added to cover the sections, incubated at 37°C for 60 min in a humidified chamber. TdT was omitted from negative control slides. To localise cells containing labelled DNA strand breaks, sections were washed and incubated with peroxidase-labelled streptavidin for 30 min, and were finally stained with DAB solutions, followed by counterstaining with haematoxylin. Approximately 1000 colon cancer cells were counted under high-power microscopy (× 400) and the percentage of TUNEL-positive tumour cells with apoptotic morphology was calculated.

### Primary cell culture of colon cancer

Cell suspensions derived from tumour samples were obtained by enzymatic digestion with medium containing 0.1%. collagenase type IA, 0.002% DNase type II and 0.05% protease type I (Sigma Chemical Co, St Louis, MO, USA). Tumour cells were isolated by adherence to plastic culture vessels for 36 h in a 5% CO_2_ incubator at 37°C. Supernatants were discarded and tumour cells were cultured in RPMI 1640 supplemented with 10% FCS in a humidified 5% CO_2_ atmosphere. Assessment of tumour cells was performed by light microscopy, electronic microscopy and immunohistochemistry for keratin.

### Assessment of anti-FasR mAb CH11-induced apoptosis

Sensitivity of primarily cultured colon cancer cells to Fas-mediated apoptosis was determined by treatment with the agonistic anti-FasR CH11 IgM monoclonal antibody (Kamiya Biomedical Co, Thousand Oaks, CA, USA) at concentrations of 10, 100, 500, and 1000 ng ml^−1^, or isotype control IgM (Kamiya) at a concentration of 1000 ng ml^−1^. In some experiments, cells were pretreated with 20 mM of synthetic Ac-SLV tripeptide (Calbiochem-Novabiochem, La Jolla, CA, USA), an inhibitor of FAP-1, for 12 h, and were subsequently incubated with anti-FasR CH11 MoAb.

After 24 h of incubation, the number of apoptotic primarily cultured colon cancer cells was determined by TUNEL assay (Boehringer Mannheim GmbH) and flow cytometry. Briefly, 1 × 10^6^ cells were washed in PBS and permeabilised with 0.1% Triton X-100 in 0.1% sodium citrate solution on ice for 5 min. Cells were washed again and incubated for 60 min at 37°C in a labelling TUNEL reaction mixture containing TdT reaction buffer (10.0 *μ*l), Br-dUTP (8.0 *μ*l), dH_2_O (32.25 *μ*l), and TdT (0.75 *μ*l). The reaction was then terminated by the addition of a rinse buffer. Cells were washed before resuspension in 0.1 ml of rinse buffer. Incorporated Br-dUTP was detected after the addition of fluorescein-labelled anti-bromodeoxyuridine antibody (5.0 *μ*l) and incubation for 30 min at room temperature in darkness. Flow cytometric analysis was performed with a FACScan flow cytometer with LYSIS II software (Nippon Becton Dickinson, Tokyo, Japan).

### Immunofluorescence analysis for FAP-1

After harvesting by scraping, 1 × 10^6^ primarily cultured colon cancer cells were washed with PBS and fixed with 1% formaldehyde for 15 min on ice. Fixed cells were centrifuged, resuspended in PBS and mixed 1 : 10 with ice-cold 70% ethanol to solubilise membranes. Then, cells were incubated with 10 *μ*g ml^−1^ polyclonal goat anti-human FAP-1 antibody (Santa Cruz Biotechnology) for 30 min at 4°C and washed in PBS. Fluorescein-conjugated anti-goat IgG, as secondary antibody (Dako Corp., Carpinteria, CA), was added to the cells for 30 min at 4°C. Cells were washed again in PBS and the intensity of fluorescence was analysed with FACScan flow cytometer and LYSIS II software (Nippon Becton Dickinson, Tokyo, Japan). An isotype-matched control antibody was used to determine nonspecific binding. A total of 10 000 cells were examined for each evaluation. Data were expressed as relative fluorescence intensity (RFI=mean fluorescence intensity of cells stained with anti-FAP-1 Ab/mean fluorescence intensity of cells stained with control Ab).

### RT–PCR detection of FAP-1

RNA was isolated from primarily cultured colon cancer cells by lysis in guanidine thiocyanate (Sigma Chemical Co, St Louis, MO, USA) followed by phenol extraction and ethanol precipitation. cDNA was synthesised from 2 *μ*g of total isolated RNA in a 20 *μ*l reaction mixture containing 4 *μ*l of 5 × RT reaction buffer, 10 U of Rnasin (Promega Corp., Madison, WI, USA), 200 *μ*M deoxynucleotide triphosphate, 40 pM oligodeoxythymidylic acid primer, and 20 U of Moloney murine reverse transcriptase (Promega). The mixture was incubated at 42°C for 1 h and then incubated at 53°C for 30 min. The unhybridised RNA was then digested with 10 U of Rnase H at 37°C for 10 min.

PCR was performed on cDNA using primers (sense and antisense) for the amplification of FAP-1 and a house-keeping gene, *β*-actin. The primers were designed according to published sequences: FAP-1, (sense) 5′-AGGTCTGCAGAGAAGCAAGAATAC-3′ and (antisense) 5′-GAATACGAGTGTCAGACATGG-3′ (Irie *et al*, 2001); *β*-actin, (sense) 5′-ACTACCTCATGAAGATCCTCA-3′ and (antisense) 5′-CAGGAGGAGCAATGATCTTGA-3′(Irie *et al*, 2001).

Thermal cycling was performed as follows: denaturation at 94°C for 1 min, annealing at 55°C for 1 min, and extension at 72°C for 1 min. In all, 30 cycles were performed for the FAP-1 PCR and 28 cycles for the *β*-actin PCR. Primers were used at a final concentration of 0.1 *μ*M each, dNTPs at 50 *μ*M, MgCl_2_ at 1.5 mM, *Taq* DNA polymerase at 1.0 U per 50 *μ*l reaction. PCR products of 607 and 762 bp were predicted for FAP-1 and *β*-actin, respectively. PCR products were separated by electrophoresis in 2% agarose gels and stained with ethidium bromide. The target bands were analysed densitometrically by using a GS-700 Imaging Densitometer (BioRad, Hercules, CA, USA). Fas-associated phosphatase-1 cDNA was semiquantitated by densitometric comparison with *β*-actin from the same sample.

### Statistical analysis

All experiments for cell cultures were performed at least three times. The results are described as mean±standard deviation. Statistical analysis was performed by one-way analysis of variance and comparisons among groups were performed by independent sample *t*-test or Bonferroni's multiple-comparison *t*-test. To examine the correlation between the number of FasL-positive TILs and the percentage of *in situ* apoptotic colon cancer cells, log transformation was performed on the former data, which allowed statistical analysis of a linear relationship between the two variables by calculating the Pearson's correlation coefficient. A two-tailed *P*-value less than 0.05 was considered to be statistically significant.

## RESULTS

### Immunohistochemical analysis of FAP-1, FasR, and FasL

The immunostaining with each antibody was judged to be antibody specific by using the immunising peptide for each antibody as an internal control. Inclusion of the soluble peptide immunogen during immunohistochemistry resulted in direct competitive displacement of positive staining in consecutive control sections.

**Figure 1:**
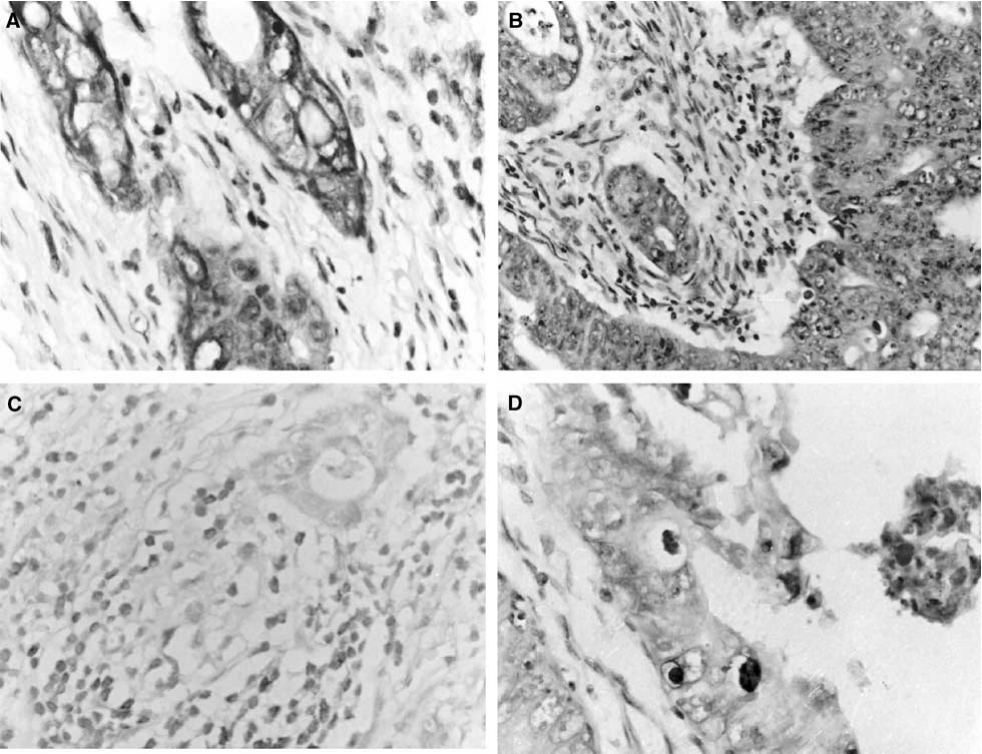
Immunohistochemical examination of FAP-1, FasR and FasL expression and TUNEL staining in human colon adenocarcinomas. Antibodies were detected using a DAB method that produces a brown colour. Counterstaining of nuclei with haematoxylin (blue). (**A**) FAP-1-positive immunohistochemical staining in the cytoplasm of colon adenocarcinoma cells. (**B**) FasR-positive immunohistochemical staining in the cytoplasm of colon adenocarcinoma cells. (**C**) FasL-positive immunohistochemical staining in the cytoplasm of TILs. (**D**) TUNEL staining in apoptotic colon adenocarcinoma cells. Only those cells with positive TUNEL staining and exhibiting apoptotic morphology were considered apoptotic.

By immunohistology, FAP-1 was present in 20 out of 28 cases (71.4%) of colon adenocarcinomas. Fas-associated phosphatase-1 was primarily localised in carcinoma cells, rather than in infiltrating lymphocytes. Fas-associated phosphatase-1 was located primarily in the cytoplasm and along the cell membrane, while nuclei were clearly negative (Figure 1A

). Fas-associated phosphatase-1-positive cells were randomly distributed within a section. Intensity of immunostaining varied from weak to intense in neoplastic areas, both within individual tumours and among tumours, and was independent of histological type and staging of the cancer.

In contrast, FasR was present in all colon adenocarcinomas tested (Figure 1B). Extent and intensity of the staining did not vary among different tumour staging or histological types, and did not correlate to the percentage of TUNEL-positive tumour cells.

As demonstrated by CD45 immunohistochemical staining, all 28 carcinomas were infiltrated by immunocytes with lymphoid morphology. In all cases of colon carcinomas, FasL immunostaining was observed in tumour infiltrating lymphocytes (TILs) that had been identified by CD45 staining in consecutive sections (Figure 1C). The number of FasL-positive TILs observed under high-power microscopy did not correlate with cancer staging or differentiation. Tumour cells were FasL positive in 17 out of 28 (60.7%) colon carcinomas (not shown).

### Apoptosis of colon cancer cells

TUNEL-positive cancer cells were randomly distributed in tumour nests and were accompanied by morphological signs of apoptosis, such as chromatin condensation, detachment of the cytoplasm from the environment and formation of apoptotic bodies (Figure 1D). TUNEL staining was also observed in TILs near or distal to tumour nests.

**Figure 2:**
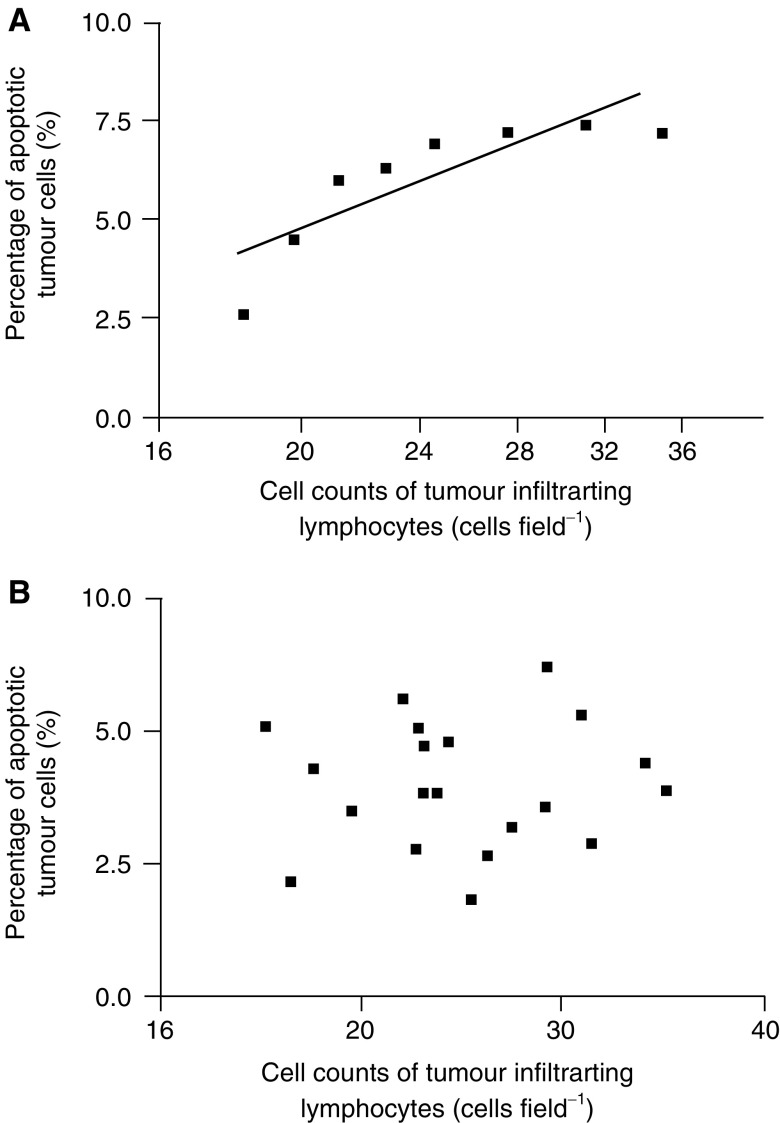
Correlation between the percentage of *in situ* apoptotic colon adenocarcinoma cells and the number of FasL-positive TILs. (**A**) In FAP-1-negative colon cancers, a linear correlation was observed between the percentage of apoptotic tumour cells and the log value of FasL-positive TIL counts (*r*=0.835, *P*<0.01). (**B**) In FAP-1-positive colon cancers, the percentage of apoptotic tumour cells was not correlated to FasL-positive TIL counts.

In colon cancers with negative FAP-1 immunostaining (*n*=8), a linear correlation was observed between the percentage of apoptotic cancer cells and the log value of Fas-positive cell counts of TILs (*r*=0.835, *P*<0.01; Figure 2A

). In contrast, in FAP-1-positive colon carcinomas (*n*=20), no correlation was observed between apoptosis of cancer cells and FasL expression in TILs (Figure 2B). Additionally, the percentage of apoptotic cancer cells was lower in colon cancers with positive FAP-1 immunostaining than in FAP-1-negative tumours (4.0±1.2 *vs* 5.9±1.7; *P*<0.05). However, apoptosis of colon cancer cells did not vary with tumour stage or differentiation.

### Fas-mediated apoptosis in primarily cultured cancer cells

**Figure 3:**
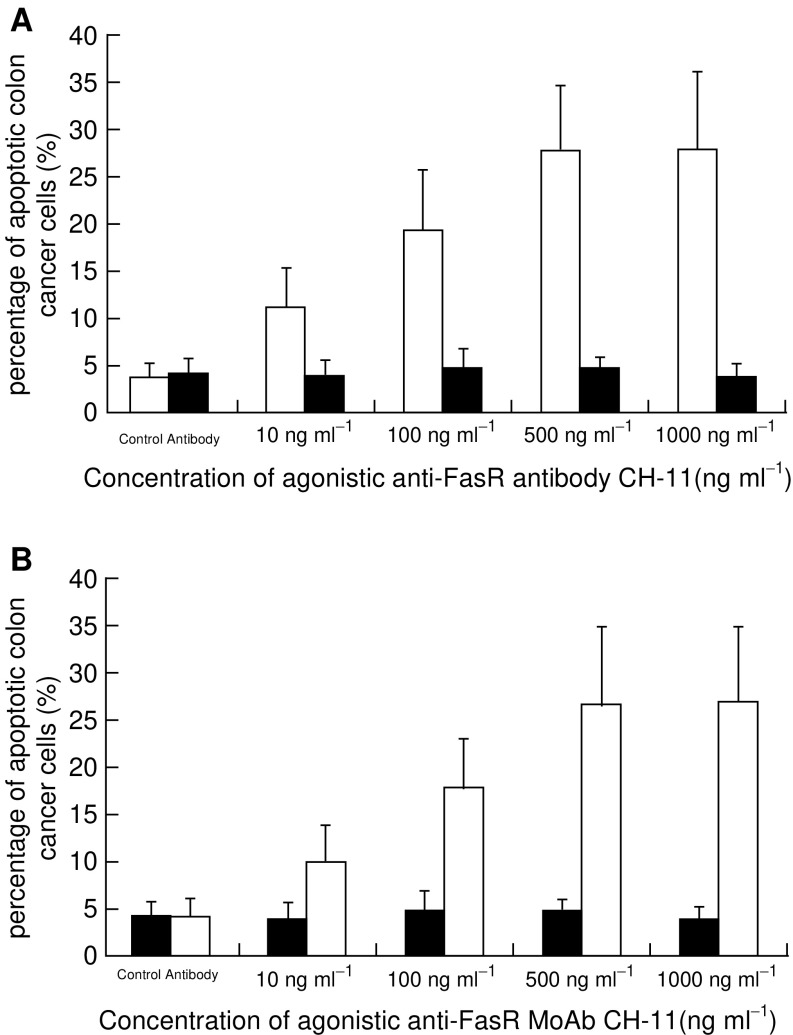
Percentage of apoptotic cultured colon cancer cells in the presence of different concentrations of CH-11. (**A**) When the concentration of CH-11 increased up to 500 ng ml^−1^, a dose-dependent increase of the apoptotic percentage of cultured colon cancer cells was observed in those cases without FAP-1 expression (□, Fas-sensitive group; *n*=8, *P*<0.05), while the ones in the cases with FAP-1 expression (▪, Fas-refractory group; *n*=20) remained stable (*P*>0.05). However, further elevation of CH-11 concentration from 500 to 1000 ng ml^−1^ did not result in a further increment of tumour cell apoptosis in Fas-sensitive group (*P*>0.05). (**B**) After preincubation with Ac-SLV, cultured colon cancer cells of Fas-refractory group showed a dose-dependent increase of the apoptotic percentage with the increase of CH-11 concentration up to 500 ng ml^−1^ (▪) (*P*<0.05) in contrast to untreated ones (□). Further elevation of CH-11 concentration from 500 to 1000 ng ml^−1^ did not result in a further increment of tumour cell apoptosis (*P*>0.05). Ac-SLV alone did not increase apoptosis of tumour cells when treated with control antibody (*P*>0.05).

To further evaluate the sensitivity of colon cancer cells to FasR-mediated apoptosis, primarily cultured tumour cells from each resected sample were individually treated with agonistic anti-Fas MoAb CH-11. Purity of isolated cancer cells, as assessed by light microscopy, electronic microscopy and immunohistochemistry for keratin, was more than 97% in each case (not shown). After incubation for 24 h in the presence of CH-11 at concentrations of up to 500 ng ml^−1^, a dose-dependent increase of apoptotic cells was observed in cultured tumour cells of the eight cases of colon cancers without FAP-1 immunostaining (FasR-sensitive group) (Figure 3A

).

The percentage of cell death was elevated to 11.19±4.04 at 10 ng ml^−1^ of CH-11, which was significantly higher with control antibody (*P*<0.05), and reached 27.63±7.08 at a concentration of 500 ng ml^−1^ of CH-11. However, higher doses of CH-11 (to 1000 ng ml^−1^) did not result in a further increase of tumour cell apoptosis (*P*>0.05). In contrast, the percentage of apoptotic tumour cells in the other 20 cases of colon cancers with FAP-1 immunostaining remained stable despite increasing CH-11 concentrations (FasR-refractory group). Even in the presence of 1000 ng ml^−1^ CH-11, the percentage of apoptotic cancer cells in this group was 3.82±1.36, which was not significantly different from treatment with control antibody (4.12±1.53; *P*>0.05).

### FAP-1 expression levels in colon cancer cells

**Figure 4:**
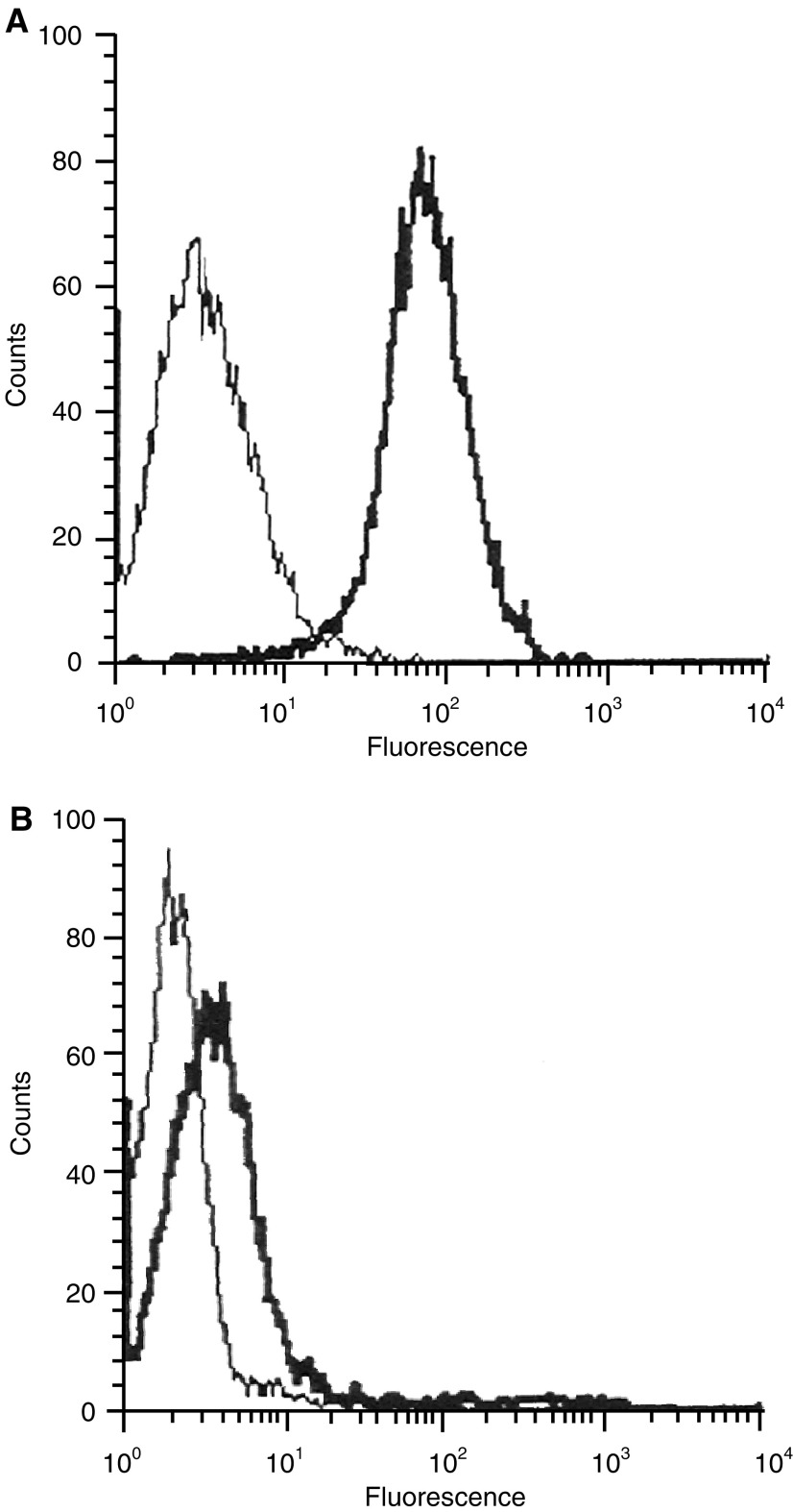
Flow cytometric analysis of FAP-1 expression on cultured colon cancer cells of Fas-refractory group (**A**) and Fas-sensitive group (**B**) using polyclonal anti-FAP-1 antibody (bold peaks) or an irrelevant antibody (isotype control) for the determination of background staining (thin-line peaks).

**Figure 5:**
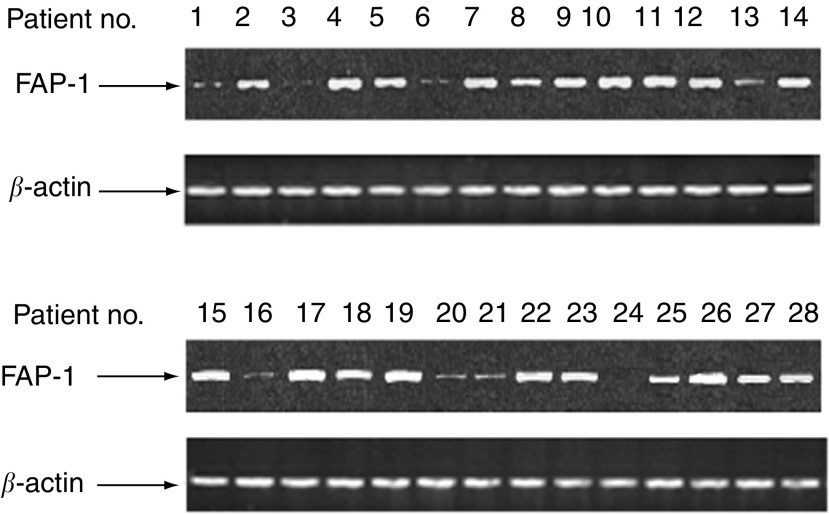
RT–PCR analysis of FAP-1 mRNA expression in cultured colon cancer cells. Patients no. 1, 3, 6, 13, 16, 20, 21, and 24 belonged to the Fas-sensitive group (sensitive to CH11-induced apoptosis; *n*=8), while the rest belonged to Fas-refractory group (resistant to CH11-induced apoptosis; *n*=20).

To confirm the difference of FAP-1 expression levels between the FasR-sensitive and FasR-refractory group, we quantified protein and mRNA expression of FAP-1 in cultured cancer cells by flow cytometric immunofluorescence analysis and RT–PCR. As shown by flow cytometric immunofluorescence analysis, FAP-1 protein was strongly expressed by colon cancer cells of the FasR-refractory group (*n*=20; RFI=38.62±5.82), which showed positive immunostaining with FAP-1 antibody in tissue sections (Figure 4A

). In contrast, only a limited amount of FAP-1 protein was detected in tumour cells of the FasR-sensitive group (*n*=8; RFI=5.1±1.8), which was significantly lower than that of the FasR-refractory group (*P*<0.01) (Figure 4B). Similarly, FAP-1 mRNA was abundantly expressed by tumour cells from the FasR-refractory group, while a much weaker expression of FAP-1 mRNA was observed in the Fas-sensitive group (Figure 5

). Semiquantification of RT–PCR results revealed that the relative amount of FAP-1 mRNA in the Fas-refractory group (*n*=20; 1.82±0.63) was significantly higher than in the Fas-sensitive group (*n*=8; 0.37±0.19) (*P*<0.01).

### Inhibition of FasR resistance by Ac-SLV

To further investigate whether FAP-1 expression did contribute to the FasR refractory property of colon cancer cells, we blocked the interaction between FAP-1 and FasR. Before treatment with CH-11 mAb, cultured tumour cells were preincubated with Ac-SLV. Ac-SLV has the same amino-acid sequence as the C-terminal end of FasR. Thus, Ac-SLV competes with FasR for the binding to FAP-1 (Yanagisawa *et al*, 1997; Sawa *et al*, 1999), prominently enhancing FasR sensitivity of colon cancer cells in the FasR-refractory group. It also increased the number of apoptotic tumour cells after incubation with CH-11 in a dose-dependent manner (Figure 3B). The percentage of apoptotic cancer cells from the FasR-refractory group pretreated with Ac-SLV was 9.91±3.83 in the presence of 10 ng ml^−1^ CH-11, which was significantly higher than in controls (*P*<0.05), and reached 26.72±8.2 when the concentration of CH-11 was increased to 500 ng ml^−1^. However, a concentration of 1000 ng ml^−1^ of CH-11 did not increase in apoptosis of colon cancer cells any further (*P*>0.05).

On the other hand, treatment with Ac-SLV alone did not increase the apoptosis of colon cancer cells (Figure 3B).

Furthermore, preincubation with Ac-SLV did not affect the apoptosis of colon cancer cells from the FasR-sensitive group significantly in the presence of CH-11 at different concentrations (data not shown).

## DISCUSSION

The present study demonstrated a high incidence of FAP-1 expression (71%) in colon carcinomas in the presence of Fas receptors. Moreover, FAP-1-expressing colon cancer cells were refractory to FasR-mediated apoptosis. This resistance could be reversed by blockade of FAP-1 and FasR interaction with an interacting tripeptide Ac-SLV.

It has been demonstrated *in vitro* and *in vivo* that colon cancer cells coexpress Fas receptors and functional FasLs on their cell surface (Houghton *et al*, 1997; Ding *et al*, 1998; Krammer *et al*, 1998; O'Connell *et al*, 1998; Peduto *et al*, 1999; Yoong *et al*, 1999; Vermijlen *et al*, 2001). Thus, it was not surprising that Fas receptor was expressed in all cases of colon carcinomas to some extent. Approximately 61% of colon cancers coexpressed FasL. A recent study has also reinforced the notion that colon cancers are resistant to FAS-mediated apoptosis (Houston *et al*, 2003). Furthermore, like in other malignancies (O'Connell *et al*, 1999; Fukuzawa *et al*, 2001; Meech *et al*, 2001), FasL-positive TILs were found in all cases of colon cancer in our study, which probably represented the local immune response of hosts against tumours. As activated CTLs and NK cells can express FasL and induce apoptosis in Fas-bearing cells, it is unclear as to why Fas-bearing cancer cells are not destroyed by TILs, which express FasL, and why colon cancer cells do not undergo apoptosis via Fas and FasL interaction.

Presently, the exact mechanisms by which colon cancer cells escape from FasL-induced apoptosis are not fully understood. Some authors hypothesised that colon cancer cells are resistant to FasR-mediated apoptosis due to the presence of inhibitory proteins in the Fas signal transduction pathway (Ding *et al*, 1998; Krammer *et al*, 1998; O'Connell *et al*, 1998; Yoong *et al*, 1999; Vermijlen *et al*, 2001). A possible candidate is FAP-1 because activation and dysregulation of the FAP-1 promoter has been demonstrated in human colon carcinoma cell line *in vitro* (Irie *et al*, 2001). We examined this hypothesis by investigating the role of FAP-1 as an inhibitor in FasR-mediated apoptosis of colon cancer cells.

Using immunohistochemistry, we demonstrated FAP-1 expression in more than 70% of colon cancers, concurring with the immunohistochemical localisation of FAP-1 in a large panel of cancers of various origins, including colon, lung, and breast carcinomas, reported by Lee *et al* (1999b). Additionally, FAP-1 immunostaining, herein, was observed along the cell membrane and/or in the cytosol, suggesting a possible role for FAP-1 to interact with the cytosolic domain of Fas just beneath the cell membrane (Ungefroren *et al*, 1999). This hypothesis was further supported by the present findings that colon cancers with FAP-1 expression had fewer apoptotic tumour cells in comparison to the ones without such expression. Furthermore, in colon cancers with FAP-1 expression, the percentage of apoptotic colon cancer cells, which bore FasR on their surface, did not correlate with the number of FasL-positive TILs surrounding the tumour nests. In contrast, the percentage of apoptotic colon cancer cells in tissue sections revealed a positive correlation with the FasL-positive TIL counts. The correlation was more pronounced when the level of FasL-positive TIL counts was in the lower range. This suggests that the sensitivity of colon cancer cells to FasR-mediated apoptosis depends on *in vivo* FAP-1 expression. *In vivo* expression of FAP-1 was also suggested to be a possible mechanism for Fas resistance in human hepatoblastomas by Lee *et al* (1999b).

Although FAP-1 has been suggested to interact with the ‘suppressive domain’ of Fas receptor that is involved in the inhibition of the apoptotic signal (Ungefroren *et al*, 2001), no direct proofs exists for the contribution of FAP-1 expression to FasR resistance in colon cancer cells. Hence, we performed primary cultures of colon cancer cells on our samples, and treated them with CH11, an agonistic Fas mAb, in order to demonstrate directly the influence of FAP-1 expression on FasR sensitivity. In consistence with our *in vivo* evaluation, our data demonstrated a dose-dependent increase in the amount of apoptosis induced by CH-11 in colon cancer cells in parallel to a reduced expression of FAP-1 mRNA and protein.

On the other hand, despite exposure to a high dose of CH-11 (1000 ng ml^−1^), colon cancer cells, which had pronounced FAP-1 expression at mRNA and protein levels, were irresponsive to FasR-mediated apoptosis. These data are in line with a recent finding on a pancreatic cancer cell line, showing that FAP-1-expressing pancreatic cancer cells were resistant to Fas-mediated apoptosis (Ungefroren *et al*, 1999).

Furthermore, treatment with Ac-SLV tripeptide abrogated the resistance of Fas-mediated apoptosis in FAP-1-expressing colon cancer cells, as apoptosis was induced in these cells by CH-11 in a dose-dependent manner. Ac-SLV shares the C-terminal three amino acids (SLV) with FasR. This sequence is necessary and sufficient to interact with FAP-1 (Moulian *et al*, 1999). Thus, Ac-SLV can competitively prevent the interaction of FAP-1 with Fas and results in the induction of Fas-mediated apoptosis (Yanagisawa *et al*, 1997; Sawa *et al*, 1999). Similarly, application of Ac-SLV in thyroid follicular cells has also been found to enhance Fas-mediated apoptosis (Myc *et al*, 1999). In this context, the present inhibition of Ac-SLV of FasR resistance in colon cancer cells substantiates our hypothesis that FAP-1 is an inhibitor of Fas-mediated apoptosis in colon cancer cells.

Interestingly, in colon cancers without FAP-1 expression, the percentage of apoptotic tumour cells was positively correlated to the logarithmic value of FasL-positive TIL count.

Likewise, even when the interaction between FasR and FAP-1 was blocked, agonistic anti-Fas mAb enhanced apoptosis in primarily cultured tumour cells in a dose-dependent manner up to a concentration of 500 ng ml^−1^. This suggests that, in addition to FAP-1 expression, there are other factors in the Fas signalling pathway that can influence celluar sensitivity to Fas-mediated apoptosis in colon cancer cells, for example, reduction of Fas receptor levels on colon cancer cells (Butler *et al*, 2000; Song *et al*, 2001b), release of soluble Fas (Cheng *et al*, 1994) or decoy receptor (Pitti *et al*, 1998) for FasL that blocks FasL on CTLs, mutation or modification of an essential signalling molecule of the Fas signaling pathway (Itoh and Nagata, 1993), and increased expression of BCL-2 gene (Meterissian and Kontogiannea, 1996). Hence, further studies are needed to elucidate the complete pathway of Fas signaling processes in generating apoptotic responses in colon carcinomas.

In conclusion, the present study suggests that FAP-1 expression contributes to the resistance of colon cancer cells to Fas-mediated apoptosis and, thus, is involved in immune escape from CTLs and NK cells. These effects may be abolished by a blockade of the interaction of FAP-1 with FasR.
